# Effectiveness of BNT162b2 Vaccine Against Symptomatic SARS-CoV-2 Infection in Children Aged 5–11 Years in Japan During Omicron Variant Predominate Periods

**DOI:** 10.2188/jea.JE20230093

**Published:** 2024-05-05

**Authors:** Megumi Hara, Yuko Ohta, Naoki Fusazaki, Yoshio Hirota

**Affiliations:** 1 Department of Preventive Medicine, Faculty of Medicine, Saga University, Saga, Japan; 2 Ohta Yuko Children’s Clinic, Saga, Japan; 3 Clinical Epidemiology Research Center, SOUSEIKAI Medical Group (Medical Co. LTA), Fukuoka, Japan

**Keywords:** coronavirus disease 2019, severe acute respiratory syndrome coronavirus 2, Omicron, vaccine, child

## Abstract

**Background:**

Although the effectiveness of BNT162b2 messenger RNA vaccines against the Omicron variant has been reported in several countries, data are limited in children living in Asian countries. Therefore, this study aimed to estimate the effectiveness of the pediatric primary two-dose monovalent mRNA vaccine series in preventing symptomatic novel coronavirus disease 2019 (COVID-19) in Japan.

**Methods:**

We conducted a test-negative case-control study (262 test-positive cases and 259 test-negative controls) in patients aged 5–11 years who presented with COVID-19-like symptoms during the Omicron BA.2- and BA.5-predominant periods. Vaccination status, demographic data, underlying medical conditions, lifestyle, personal protective health behaviors, living environment, and PCR test results were obtained using parent-administered questionnaires and clinical records. Vaccine effectiveness (VE) against symptomatic COVID-19 was calculated using a multivariate logistic regression analysis.

**Results:**

Of the test-positive cases and test-negative controls, 9.2% (*n* = 24) and 12.7% (*n* = 33) received two vaccine doses, respectively. Having siblings and a BA.5-dominant period were significantly associated with symptomatic COVID-19. After adjusting for age, siblings, study period, and duration after the last vaccination, the overall VE of two-dose vaccination was 50.0% (95% confidence interval [CI], 5–74%). VE was 72% (95% CI, 24–89%) within 3 months after the two-dose vaccination, while it decreased to 24% (95% CI, −80% to 68%) after 3 months.

**Conclusion:**

Two BNT162b messenger RNA vaccine doses provided moderate protection against symptomatic COVID-19 during the Omicron variant period. A time-dependent decrease in VE was noted after the second dose; thus, a booster dose 3 months after the second dose is warranted.

## INTRODUCTION

The BNT162b2 messenger RNA (mRNA) monovalent vaccine (Pfizer, New York; BioNTech, Mainz, Germany) against novel coronavirus disease 2019 (COVID-19) has been authorized for use in 5–11-year-old children with antigen doses of 10 mg. Vaccination for this group began in March 2022, when the Omicron variant (B.1.1.529) was predominant in Japan. Because the epidemic status or severity of infection with the Omicron variant in pediatric patients was not definitive and the evidence regarding vaccine effectiveness (VE) against the Omicron variant in preventing the onset and severity of illness in pediatric patients was insufficient, mandatory vaccination was not applied to children of the aforementioned age group at the beginning.^1^ However, in September 2022, although the proportion of severe cases and deaths was low and moderate efficacy against the onset of Omicron variant and high efficacy against hospitalization had been reported, vaccination to this age group was encouraged in an endeavor to control the rising number of infected cases with the Omicron variant.^2^^–^^6^ Yet, vaccination coverage in this age group remains low; as of January 2023, only approximately 20% have been vaccinated.^7^ The following reasons for non-vaccination have been reported: novelty, lack of information, and lack of confidence in VE.^8^^,^^9^

Regarding the effectiveness of the primary two-dose mRNA vaccine series in adults, VE against the Omicron variant has been reported to be lower than that against the Delta and Alpha variants, likely because of its high transmissibility and immune evasion.^10^^,^^11^ In addition, the rapid-waning of two-dose mRNA VE against symptomatic Omicron variant infection has been reported.^12^ Similar to the results in adults, a recent meta-analysis of 17 studies conducted in children aged 5–11 years reported that a two-dose mRNA vaccine is associated with lower risks of SARS-COV-2 infections with or without symptoms, severe COVID-19-related illness, and hospitalization.^13^ However, reports on VE in children are still limited, the duration after the second dose is short, and no studies have been reported in Japan.

The Omicron lineage of SARS-CoV-2 continues to evolve; the predominant sublineage was BA.2, from March to June, and evolved to BA.5 after July; these sublineages caused the sixth and seventh waves in Japan, respectively.^14^ Laboratory studies have reported that BA.5 is more transmissible and more evasive to antibodies than BA.2.^15^^–^^17^ A recent observational study in adults showed a lower overall VE estimate for three monovalent mRNA doses during the BA.4/BA.5 period than during the BA.1/BA.2 period.^18^ In contrast, studies from South Africa and the United Kingdom showed no difference in the VE of three monovalent mRNA vaccine doses against hospitalization during the BA.4/BA.5 period compared with the BA.1/BA.2 period at the same vaccination intervals.^19^^,^^20^ To the best of our knowledge, no study has compared VEs against BA.2 and BA.5 among children.

The aim of this study was to estimate the effectiveness of the pediatric primary two-dose monovalent mRNA vaccine series in preventing symptomatic COVID-19 during the predominant Omicron variant period in Japan.

## METHODS

### Study design

We conducted a test-negative case-control single-center study on children in Saga City, southern Japan. Inclusion criteria were patients aged 5–11 years who visited a pediatric clinic that provided standalone community/primary care healthcare services, those who did not have a COVID-19 history, and those with COVID-19-like symptoms, including fever, headache, fatigue, cough, runny nose, wheezing, diarrhea, sore throat, appetite loss, taste disorder, and smell disorder who were examined for SARS-CoV2 reverse transcription polymerase chain reaction (PCR) assay using a nasopharyngeal swab. Patients with a positive test result were defined as cases, whereas patients with a negative test result were defined as controls. Exclusion criteria were patients who had negative test results within 14 days of the previous negative test results. When patients had multiple positive results, we only included the first episode (eFigure 1). The survey period coincided with the sixth and seventh waves of COVID-19 driven by the Omicron BA.2- and BA.5-predominant periods (1 March–30 June 2022 and 1 July–November 2022, respectively).^21^ The pediatricians explained the study purpose, contents, and conditions of cooperation to both the patients and their parents. After informed consent was obtained from both the patients and their parents, they were asked to answer the questionnaire when they were cured and send it back to the administrative office. For questionnaires that had missing information, the research nurses contacted parents by phone and requested the missing information. The study protocol was approved by the Ethics Committee of Saga University (approval no. R3-60), and written informed consent was obtained from all participants before the commencement of the study.

### Data collection

The participants’ parents answered a questionnaire on behalf of their children to provide the information. The questionnaire included questions on characteristics such as sex, date of birth, weight, height, and area of residence. Questions regarding underlying medical conditions were also included, such as bronchial asthma, food allergy, epilepsy, allergic rhinitis, atopic dermatitis, heart disease, kidney disease, and digestive disease. Additionally, history of pneumonia and general health conditions were addressed. Furthermore, parents answered questions regarding second-hand smoking history, physical activity, sleep, eating habits, frequency of going out, going to school or day-care, education, birth weight, blood type, family members living together, siblings, size of residential space, and whether there were any COVID-19-infected people in close proximity. We asked whether the child implemented any of the following personal protective health measures: mask-wearing, washing their hands for at least 20 seconds upon returning home, use of chlorine- or ethanol-based disinfectants, physical distancing, regular ventilation of the home, avoiding eating meals with five or more persons, and regularly obtaining COVID-19 information. In Japan, BNT162b2 has been used in children aged 5–11 years. Vaccinated individuals responded to a questionnaire regarding lot number, number of doses, and date of vaccination based on their vaccination certificate. The patients reported symptoms of COVID-19. PCR test results and diagnosis date were ascertained using medical records. The minimum sample size required for the study was calculated as follows: assuming α = 0.05/number of variables (20 items) = 0.0025, β = 0.20, VE = 50–70%, and the proportion of vaccination uptake among controls = 20%, the required minimum sample size was 107–260 cases and controls.

### Statistical analysis

The primary analysis assessed VE against symptomatic SARS-CoV-2 infection with one or two vaccine doses compared with no vaccination according to the BA.2 or BA.5 lineage-predominant period, as well as the entire period, which is the Omicron variant-predominant period.^14^ Subgroup analyses were performed to estimate VE according to the duration between the last vaccination date and PCR test date for all periods. To assess differences between case and control participants, background characteristics, such as sex, age group (5–6, 7–9, and 10–11 years old), comorbidities, personal protective health behaviors, siblings, using day-care or going to school, second-hand smoking, parental COVID-19 vaccination status, vaccination dose, and duration between the day of diagnosis and last vaccination day, were analyzed using the chi-square test or *t*-test. Multivariable logistic regression models were constructed to calculate odds ratios (ORs) with 95% confidence intervals (CIs). Selection of covariates was based on the univariate associations between variables and the case-control status. The included covariates were age group, use of hand sanitizer, siblings, and duration after the last vaccination for the BA.2-predominant period and age group, social distancing of 1.5 meters, siblings, and duration after the last vaccination for the BA.5-predominant period. For VE of the entire study period, age, siblings, and duration after the last vaccination were included in the model as covariates, and the study period was adjusted as a stratified variable. The adjusted VE was calculated as (1 − adjusted OR) × 100 (%). All statistical analyses were performed using SAS software version 9.4 (SAS Institute, Cary, NC, USA).

## RESULTS

In total, 577 children underwent a PCR test for SARS-CoV-2 in the clinic between March 1, and November 30, 2022. The parents of 521 children (90.3%) agreed to participated in this study; the 521 enrolled children comprised 262 cases and 259 controls. The BA.5-predominant period included more cases than the BA.2-predominant period (*P* < 0.001). The characteristics of the children according to the study period are shown in Table 1. One of the participants did not provide vaccination dose data in the questionnaire. Despite our best efforts to contact the parents, we did not receive a response. There was a significantly higher proportion of siblings among the positive cases than among the controls in both periods. During the BA.2-predominant period, there was a significantly lower proportion of children using hand sanitizers and a longer duration after the second dose of vaccine in the case group than in the control group. During the BA.5-predominant period, there was a significantly lower proportion of children keeping a 1.5-meter social distance during contact with anyone in the case group than in the control group. The most common symptoms among the positive cases were fever (87.0%), cough (66.0%), fatigue (63.7%), headache (61.5%), runny nose (55.7%), and sore throat (43.1%) (eTable 1).

**Table 1.** Characteristics of study participants during the Omicron variant according to epidemic period in Japan

		BA.2-predominant period					BA.5-predominant period				
	
		Case (*n* = 122)		Control (*n* = 188)			Case (*n* = 140)		Control (*n* = 71)		
	
		*n*	%	*N*	%	*P*-value^a^	*N*	%	*n*	%	*P*-value^a^
**Sex**	Women	53	43.4	78	41.5	0.734	61	43.6	31	43.7	0.990
**Age group, years**	5–6	40	32.8	68	36.2	0.309	55	39.3	38	53.5	0.136
	7–9	46	37.7	79	42.0		52	37.1	19	26.8	
	10–11	36	29.5	41	21.8		33	23.6	14	19.7	
**Any comorbidity** ^b^ **: Yes**		21	17.2	37	19.7		16	11.4	12	16.9	0.268
**Protective health behavior**											
** Wear a mask during contact with anyone**		110	90.2	170	90.4	0.939	116	82.9	58	81.7	0.833
** Wash hands for over 20 s each time**		48	39.3	83	44.1	0.403	66	47.1	33	46.5	0.927
** Use a hand sanitizer**		85	69.7	157	83.5	0.004	118	84.3	54	76.1	0.146
** Social distancing of 1.5 m during contact with anyone**		79	64.8	118	62.8	0.722	88	62.9	57	80.3	0.009
** Regular ventilation and disinfection**		92	75.4	140	74.5	0.852	107	76.4	49	69.0	0.249
**Siblings: Yes**		113	92.6	159	84.6	0.035	128	91.4	57	80.3	0.020
**Use of day-care service or go school: Yes**		25	20.5	42	22.3	0.399	31	22.1	15	21.1	0.866
**Second hand smoke: Yes**		48	39.3	79	42.0	0.640	54	38.6	24	33.8	0.498
**Both parents received COVID-19 vaccine: Yes**		14	11.5	26	13.8	0.546	12	8.6	3	4.2	0.246
**Vaccinated dose** ^c^	0	113	92.6	163	86.7	0.070	121	86.4	58	81.7	0.364
	1	3	2.5	4	2.1		0	0.0	1	1.4	
	2	6	4.9	21	11.2		18	12.9	12	16.9	
**Duration to PCR test, days**		Mean	SD	Mean	SD		Mean	SD	Mean	SD	
**Vaccination date**	1	11.3	8.4	17.8	10.6	0.434	Nc		nc		
	2	47.8	33.1	23.4	22.3	0.044	169.4	28.3	178.7	41.3	0.124

COVID-19, novel coronavirus disease 2019; PCR, polymerase chain reaction; SD, standard deviation.

^a^Chi-square test for categorical variables and *t*-test for continuous variables.

^b^Comorbidities are bronchial asthma, food allergy, epilepsy, allergic rhinitis, atopic dermatitis, heart disease, and kidney disease.

^c^Vaccination dose of one case during BA.5-predominant period was missing.

Table 2 shows the adjusted ORs and 95% CIs of the vaccine against symptomatic COVID-19. In the period-stratified analysis, ORs of two-dose vaccination were negatively associated with symptomatic COVID-19 in both the BA.2- and BA.5-predominant periods, although statistical significance was not detected. The adjusted OR during the entire period of two-dose vaccination was 0.50 (95% CI, 0.26–0.95); thus, VE was estimated to be 50% (95% CI, 5–74%). Stratification by the duration after the second dose of vaccination showed a time-dependent decrease in VE. VE against symptomatic COVID-19 was estimated to be 72% (95% CI, 24–89%) within 3 months after the second dose, whereas a protective effect was not detected after 3 months.

**Table 2.** Odds ratios of the vaccine against symptomatic COVID-19

		Case (*n* = 262)	Control (*n* = 259)	Adjusted^a^ OR	95% CI
**BA.2-predominant period 6th wave (March 1 to June 30, 2022)**					
**Vaccinated dose**	0	113	163	1	
	1	3	4	1.06	(0.22–5.12)
	2	6	21	0.40	(0.15–1.06)
**BA.5-predominant period 7th wave (July 1 to November 30, 2022)**					
**Vaccinated dose** ^b^	0	121	58	1	
	1	0	1	nc	
	2	18	12	0.54	(0.20–1.41)

**Entire period (March 1 to November 30, 2022)**					
**Vaccinated dose** ^b^	0	234	221	1	
	1	3	5	0.70	(0.16–3.10)
	2	24	33	**0.50**	**(0.26–0.95)**
**Time after second dose**					
	0–13 days	1	7	0.18	(0.17–3.17)
	14–27 days	2	9	0.23	(0.05–1.10)
	28–83 days	3	6	0.57	(0.14–2.37)
	≥84 days	18	11	0.76	(0.32–1.80)

	<3 months	6	22	**0.28**	**(0.11–0.76)**
	≥3 months	18	11	0.76	(0.32–1.80)

CI, confidence interval; COVID-19, novel coronavirus disease 2019; OR, odds ratio.

^a^Adjusted for age (category), using a hand sanitizer (for BA.2 predominant period), social distancing over 1.5 m (for BA.5 predominant period), sibling, study period (for entire period), and duration after the last vaccination.

^b^Vaccination dose of one case during BA.5-predominant period was missing.

## DISCUSSION

This prospective test-negative case-control study, which was conducted during the Omicron variant-predominant period, confirmed a moderate mRNA VE against symptomatic COVID-19 among 5–11-year-old Japanese children. We estimated that the VE of two BNT162b2 doses was 50% (95% CI, 5–74%) and found a time-dependent decrease in VE after the second dose. VE was estimated to be 72% (95% CI, 24–89%) within 3 months after the second dose; however, a protective effect was not detected 3 months after the last two doses. These results provide parents with ample evidence regarding the effectiveness of the COVID-19 vaccine, guiding them to make informed decisions regarding their child’s vaccination and encouraging them to administer booster doses to their children within 3 months after the second dose.

Systematic reviews have reported that VE or efficacy against symptomatic COVID-19 peaks within 6 weeks after the second dose and then decreases within 6 months among both adults^22^^,^^23^ and children.^13^ Thus, the duration after the second dose between studies should be considered when comparing VEs between studies. Regarding VE during the Omicron variant-predominant period among children, some studies have been reported.^2^^,^^3^^,^^6^^,^^24^ In a United States-based study, the VEs against symptomatic COVID-19 were reported as 60.1% 2–4 weeks after the second dose and 28.9% 5–8 weeks after the second dose,^2^ whereas in a study based in Israel, it was reported as 48% 1–3 weeks after the second dose.^3^ The estimated VE against symptomatic COVID-19 was higher in this study than in previous studies. We confirmed significant preventive effectiveness within 3 months of the second dose; the estimated VE was 72%. In addition, we observed that VE peaked within 2 weeks after the second dose and decreased in a time-dependent manner, although we could not detect statistical significance owing to the limited sample size. Several factors, such as host factors, including body size, genetics, and comorbidity; extrinsic factors, including previous infections; behavioral factors, including wearing masks and distancing; and environmental factors, including geographic location and predominant lineages, are considered to influence immunogenicity and might have influenced VE in this study.^25^ However, the durability of VE in this study was similar to that in other studies.^2^^,^^3^^,^^6^^,^^24^ Our study showed that the protective effect against symptomatic COVID-19 disease was not detected 3 months after the second dose, which supports the need for a booster dose using the bivalent (Original/Omicron BA.4-5) COVID-19 vaccine.

Although more transmissible and more evasive to antibodies of the BA.5 lineage compared to BA.2 in laboratory studies,^15^^–^^17^ the difference in VE according to lineage was not clear in this study. VE was estimated to be slightly higher in the BA.2-predominant period than in the BA.5-predominant period; however, the CI overlapped in this study, even after adjusting for possible confounding factors, such as protective health behaviors, siblings, and duration after the last vaccination. The results of observational studies in adults have been inconsistent.^18^^–^^20^ A recent observational study conducted in the United States reported a lower overall VE estimated for three monovalent mRNA doses during the BA.4/BA.5 period than during the BA.1/BA.2 period,^18^ whereas two independent studies conducted in South Africa and the United Kingdom did not show a difference in VE according to the predominant lineage.^19^^,^^20^

A trend toward higher VE against symptomatic COVID-19 with Omicron variants in the youngest age group (5–6 years) than in the oldest age group (10–11 years) has been reported.^3^ In contrast, such a trend was not detected in our study. Estimated VEs in the 5–6, 7–9, and 10–11 age groups were 41% (95% CI, −118% to 84%), 66% (95% CI, 10–87%), and 30% (95% CI, −88% to 74%), respectively (eTable 2). The limited sample size precluded evaluation of inverse trends in age and VE in this study; therefore, further investigations are needed.

The strength of this study is that we evaluated the VE by adjusting for possible confounding factors, including health-preventive behavior and environmental factors. Some other strengths included the fact that the vaccination status was ascertained with vaccination certificate, case definition was made by PCR results, and the study had a high participation rate. This may have led to low misclassification bias and selection bias. This study had several limitations as well. First, the sample size of this study was not enough to evaluate VE from test-negative designs in conformance with the World Health Organization guidelines.^26^ It is essential to follow these guidelines for evaluating COVID-19 VE when platforms used for VE evaluations include surveillance systems for severe acute respiratory infections, influenza-like illness, other syndromic disease surveillance in sentinel hospitals, health worker surveillance, administrative databases, and well-defined outbreaks. Although the Health Center Real-time information-sharing System on COVID-19, Vaccination Record System, and medical claim data exist in Japan, these databases are not allowed to use VE studies by individual linkage. Therefore, we conducted a test-negative design case-control study in a pediatric clinical setting with a minimal sample size. Furthermore, we could not detect statistically significant associations according to the study period, duration after the last vaccination, and age group owing to the small sample size. Second, our results only provide VE data for the primary vaccination series against the Omicron variant in Japan, and a study evaluating the booster dose is needed. However, this was the first report in Japan, where vaccination confidence and parental acceptance of vaccination for their children are low.^27^ Reasons for being unsure about vaccination for children include potential side effects and uncertainty about vaccine safety among Japanese parents.^9^ Although surveillance for safety should be continued, our results may contribute to the promotion of vaccination among children by providing evidence of the benefits of vaccination to their parents. Third, residual confounding factors might exist even after adjusting for confounding factors, such as mask-wearing and distancing. While the test-negative design helps to mitigate healthcare-seeking bias, vaccinated individuals may be less likely to seek hospital care due to the perception of protection or milder symptoms.^28^ This hospital visit bias could potentially lead to an underestimation of VE Finally, the study population was only sourced from a single hospital in Japan; therefore, the results may not be generalizable to all Japanese children.

In conclusion, among 5–11-year-old Japanese children, the estimated VE for two doses of monovalent BNT162b2 vaccine against symptomatic COVID-19 during the Omicron variant predominant period was moderate, although it waned over time. Our findings could potentially help parents, clinicians, and policymakers make informed decisions regarding the importance of vaccinating children. Further studies on VE of booster doses are warranted.

## SUPPLEMENTARY MATERIAL

eFigure 1. Study participants

eTable 1. Parents reported COVID-19 symptoms according to period

eTable 2. Odds ratios of the vaccine against symptomatic COVID-19
