# Supplementary material for: Effectiveness of BNT162b2 Vaccine Against Symptomatic SARS-CoV-2 Infection in Children Aged 5–11 Years in Japan During Omicron Variant Predominate Periods
Source: J Epidemiol. 2024 May 5;34(5):205–10. doi: 10.2188/jea.JE20230093 (PMC10999521; doi:10.2188/jea.JE20230093)
Supplement: Supplementary file 1 [file je-34-205-s001.pdf]

eFigure 1. Study participants

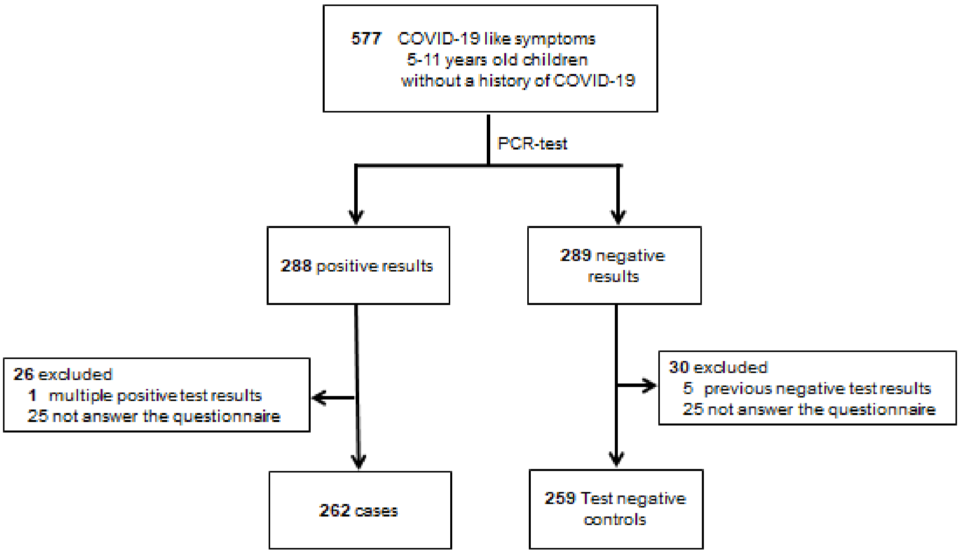

eTable 1. Parents reported COVID-19 symptoms according to period

	All duration (n=262)		BA.2 predominant period (n=122)		BA.5 predominant period (n=140)		
	n	%	n	%	n	%	*P*-value[a]
Fever ≥37.5 °C	228	87.0	100	82.0	128	91.4	0.04
Cough	173	66.0	81	66.4	92	65.7	0.91
Fatigue	167	63.7	70	57.4	97	69.3	0.06
Headache	161	61.5	68	55.7	93	66.4	0.10
Runny nose	146	55.7	66	54.1	80	57.1	0.62
Sore throat	113	43.1	43	35.2	70	50.0	0.02
Unusual muscle pains	86	32.8	36	29.5	50	35.7	0.35
Diarrhea	85	32.4	41	33.6	44	31.4	0.81
Dry eyes and mouth	42	16.0	22	18.0	20	14.3	0.52
Loss of taste or smell	29	11.1	12	9.8	17	12.1	0.75
Shortness of breath	20	7.6	10	8.2	10	7.1	0.93
Insomnia	17	6.5	10	8.2	7	5.0	0.39
Chest pain	9	3.4	5	4.1	4	2.9	0.83
Brain fog	6	2.3	1	0.8	5	3.6	0.26
Hair loss	3	1.1	1	0.8	2	1.4	0.86

COVID-19, novel coronavirus disease 2019.

[a] Frequencies were compared between periods and tested using the Chi-square test

eTable 2. Odds ratios of the vaccine against symptomatic COVID-19

		Case (n=262)	Control (n=259)	Adjusted[a] OR	95% CI
			**Entire period (March 1 to November 30, 2022)**		
**Age group**	**5–6**				
Vaccinated dose	0	89	98	1	
	1	2	1	3.16	(0.28–36.14)
	2	4	7	0.59	(0.16–2.18)
**Age group**	**7–9**				
Vaccinated dose	0	90	80	1	
	1	0	2	nc	
	2	8	16	**0.34**	**(0.13–0.90)**
**Age group**	**10–11**				
Vaccinated dose	0	56	43	1	
	1	1	2	0.54	(0.05–6.24)
	2	12	10	0.70	(0.26–1.88)

CI, confidence interval; COVID-19, novel coronavirus disease 2019; OR, odds ratio.

[a]Adjusted for age (category), siblings, study period (for entire period), and duration after the last vaccination.
